# Supplementary material for: Mechanical loading recovers bone but not muscle lost during unloading
Source: NPJ Microgravity. 2020 Dec 3;6:36. doi: 10.1038/s41526-020-00126-4 (PMC7712877; doi:10.1038/s41526-020-00126-4)
Supplement: Supplementary file 1 — Supplementary Figures and Table [file 41526_2020_126_MOESM1_ESM.pdf]

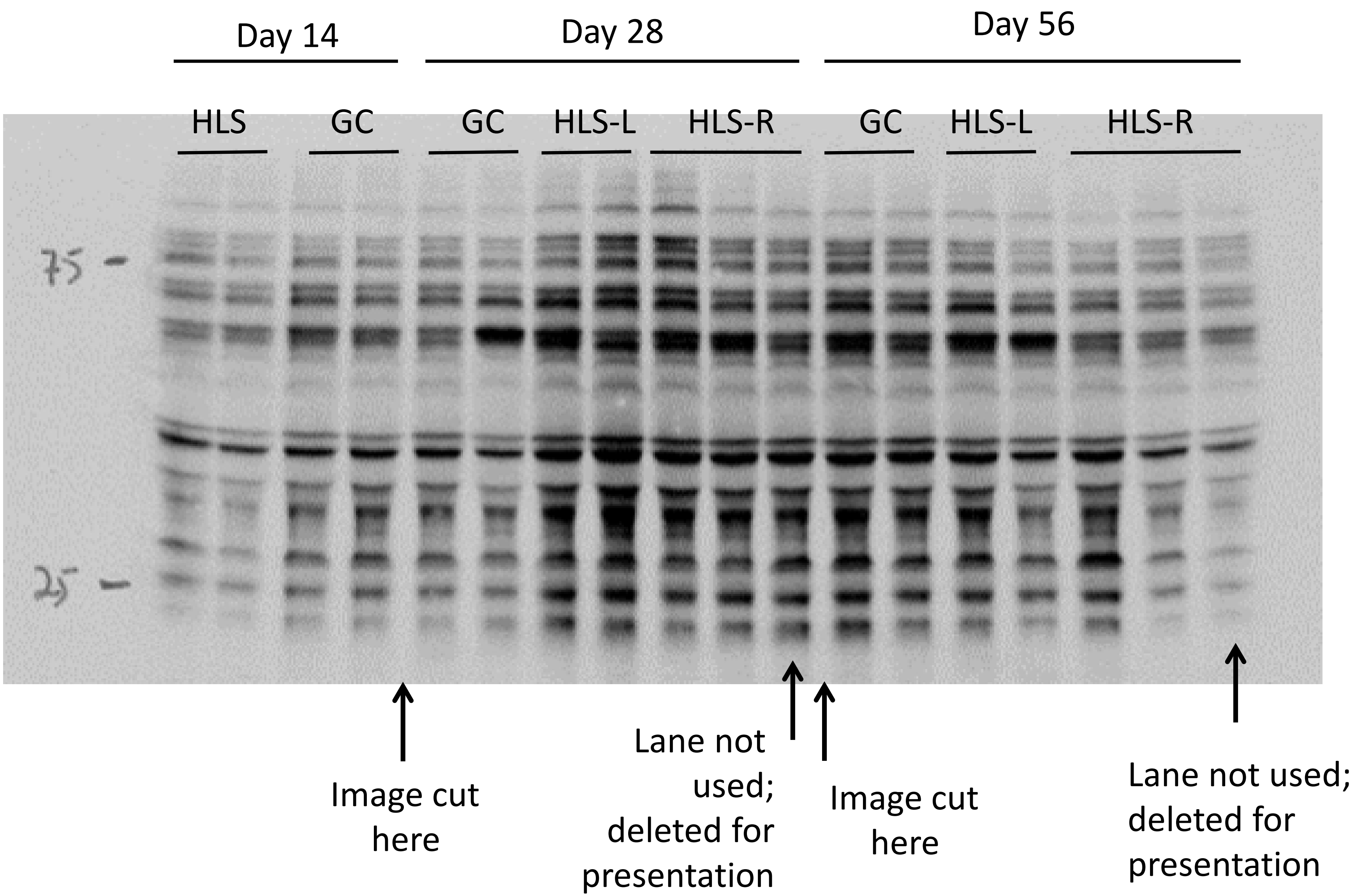

Supplementary Figure 1. Original capture of Figure 5BBecause HLS and GC samples in first 4 lanes were loaded in the wrong order; the image in these lanes was flipped 180 degrees for presentation purposes, but not otherwise altered.

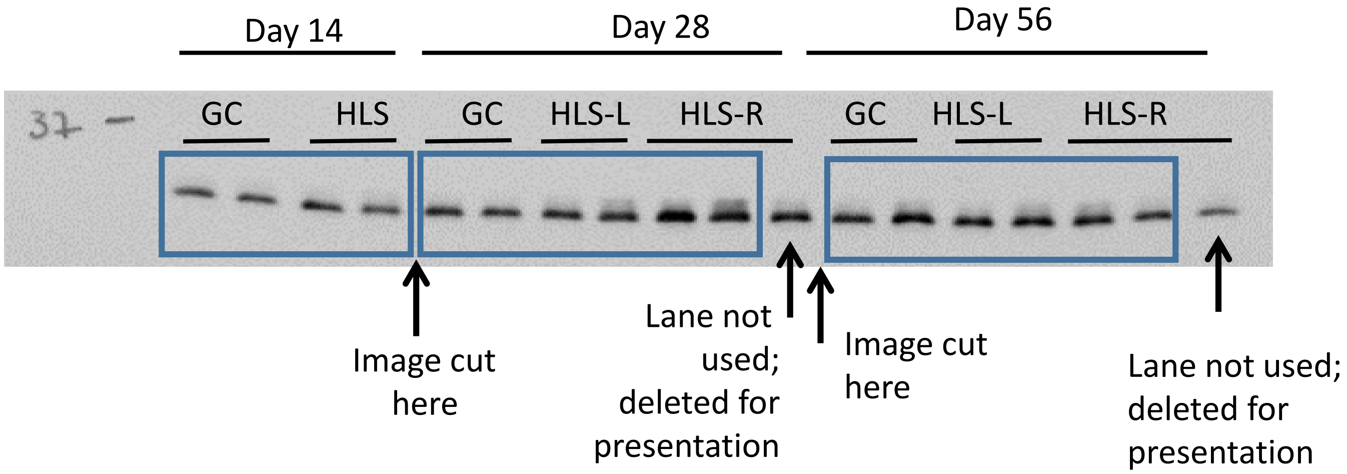

Supplementary Figure 2. Original capture of Figure 6A:S6 total. Note: For this blot and those that follow, all samples were from individual animals. Gels are shown that were used to generate each representative blot and lanes in blue boxes were used for final figures. In some cases all groups were run on the same gel; for other proteins, representative blots were obtained from different blots for each Experimental day. As statistical comparisons were performed on within day data only. Obtaining representative blots from different gels is permissible, and this information has been included in the figure legends. Individual PDVF membranes were cut so as to be able to probe for different molecular weight proteins using other antibodies.

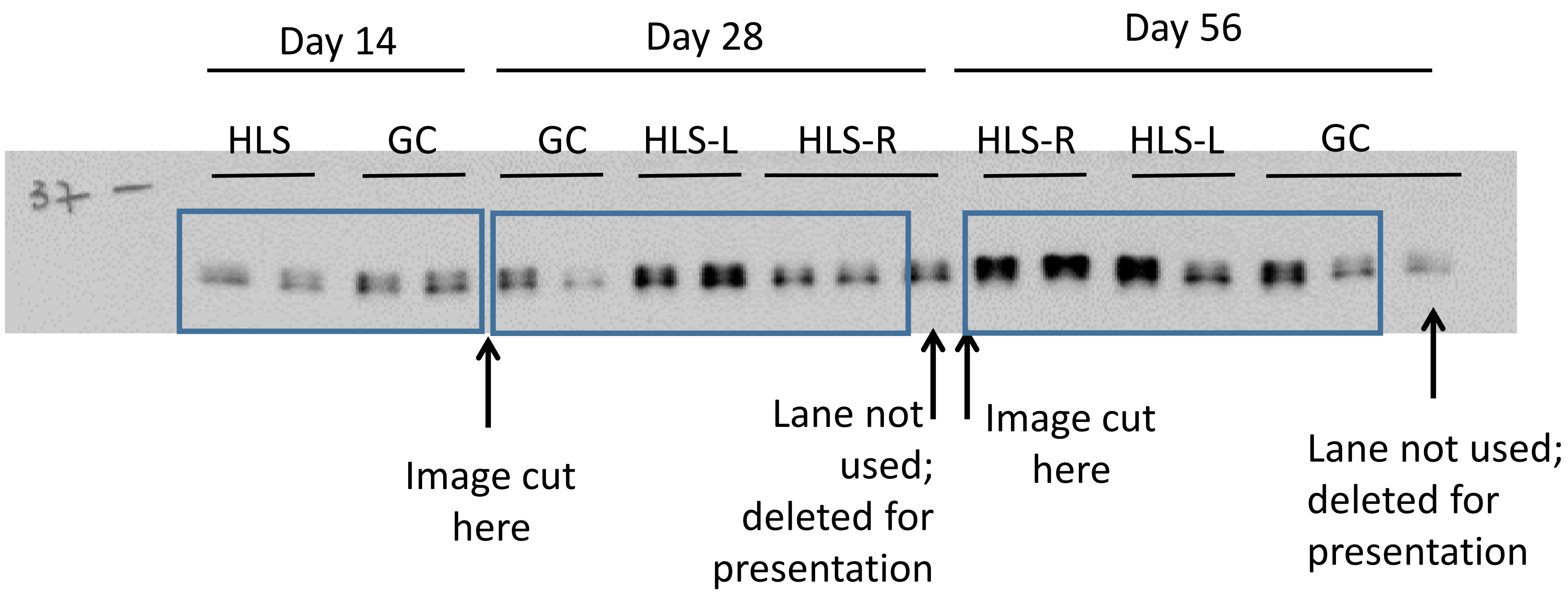

Supplementary Figure 3. Original capture of Figure 6A: S6 S240/244 phosphorylation. For presentation purposes, first 4 lanes and last 4 lanes image was reversed (flipped 180 degrees) so lanes were in the same order as bar graphs; otherwise, no other alterations. Group order was randomized and not always runin the same order to compensate for differences in any differences in transfer across the gel and to blind the technician to group identification.

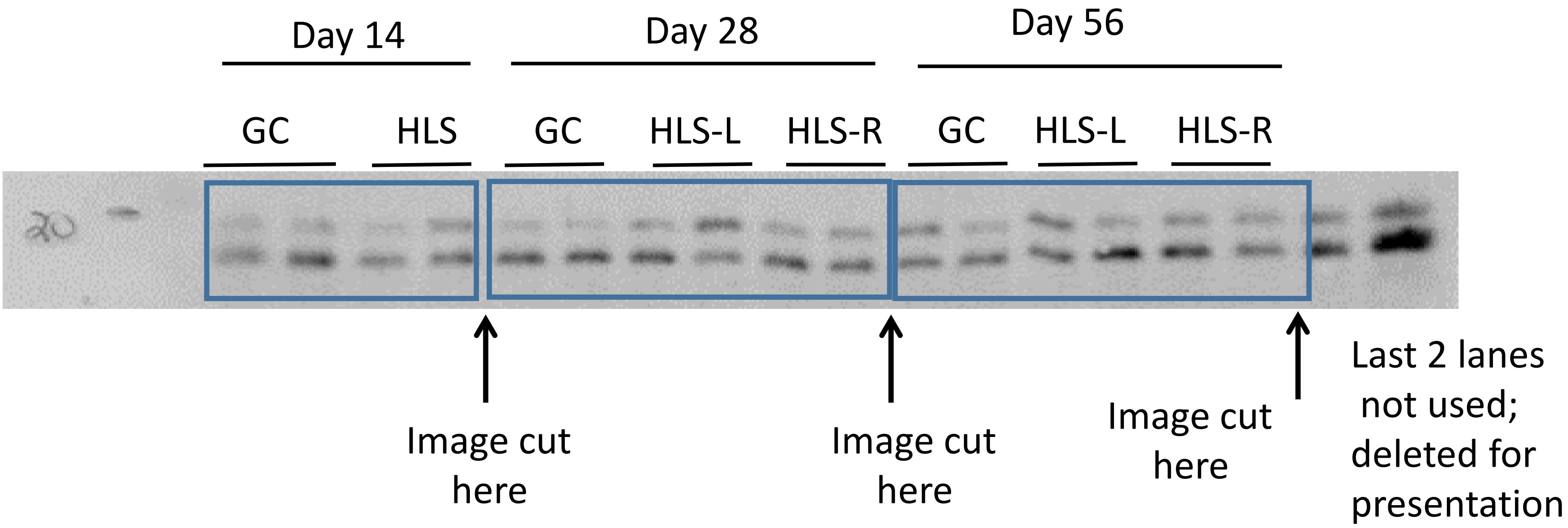

Supplementary Figure 4. Original capture of Figure 6A: 4E-BP1 – total.

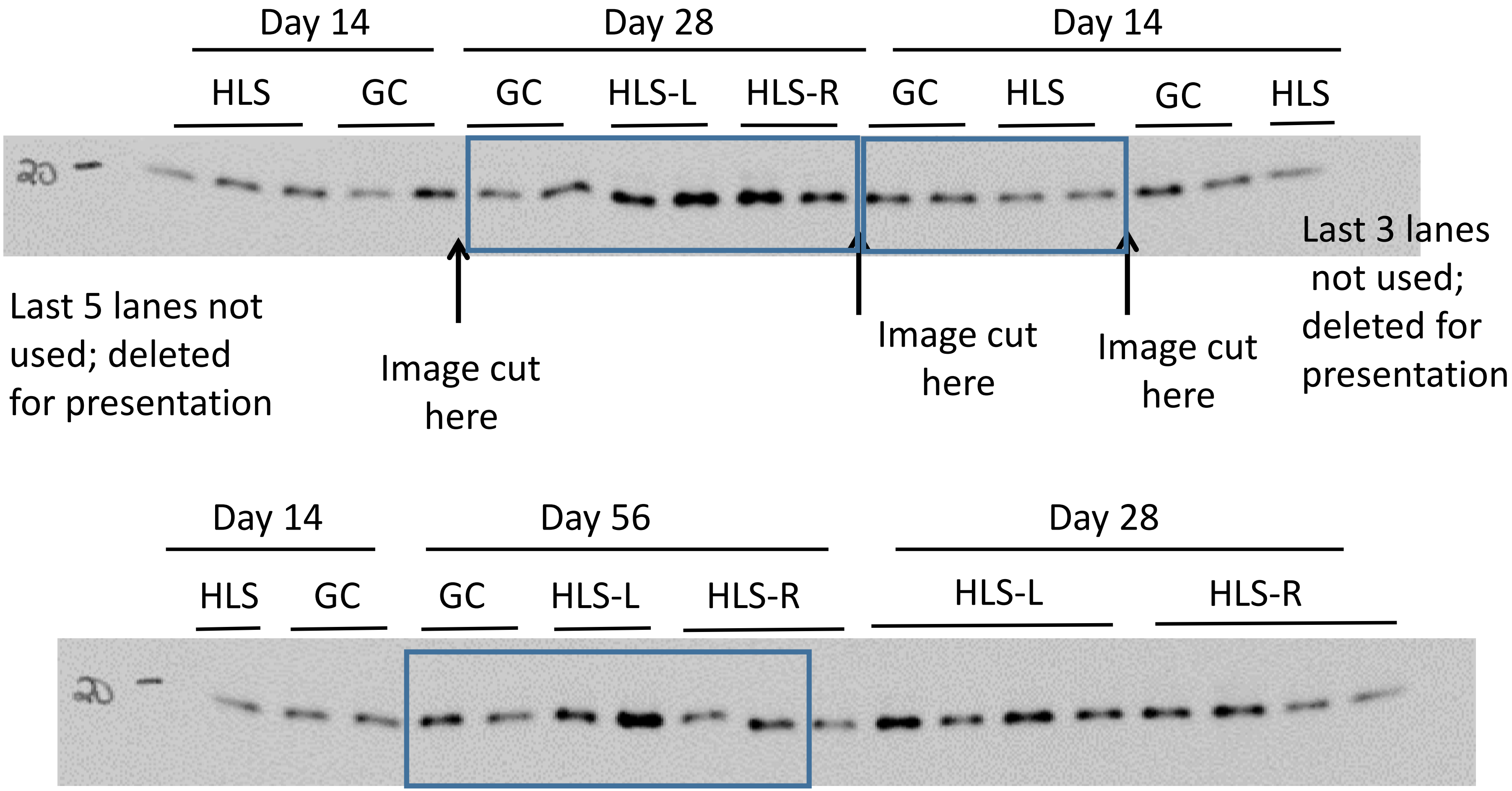

Supplementary Figure 5. Original capture of Figure 6A: 4E-BP1 S65 phosphorylated. For presentation purposes, representative blots were obtained from 2 separate gels and blots arranged to match the group order shown in respective bar graphs. All experimental groups for a specific day were obtain from the same gel. Group order was randomized and not always run in the same order to compensate for in any differences in transfer across the gel and to blind the technician to group identification. Blots were selected to best represent the overall average for a given experimental group and for each specific day.

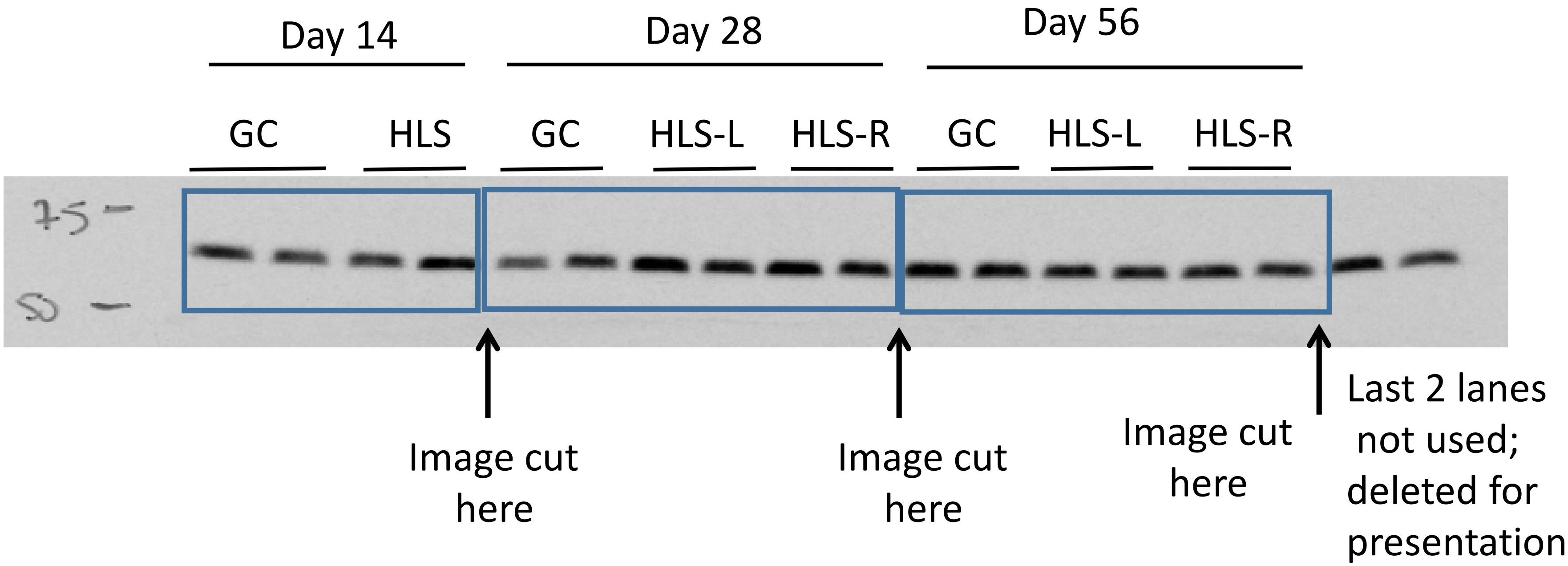

Supplementary Figure 6. Original capture of Figure 6A: S6K1 – tota.l

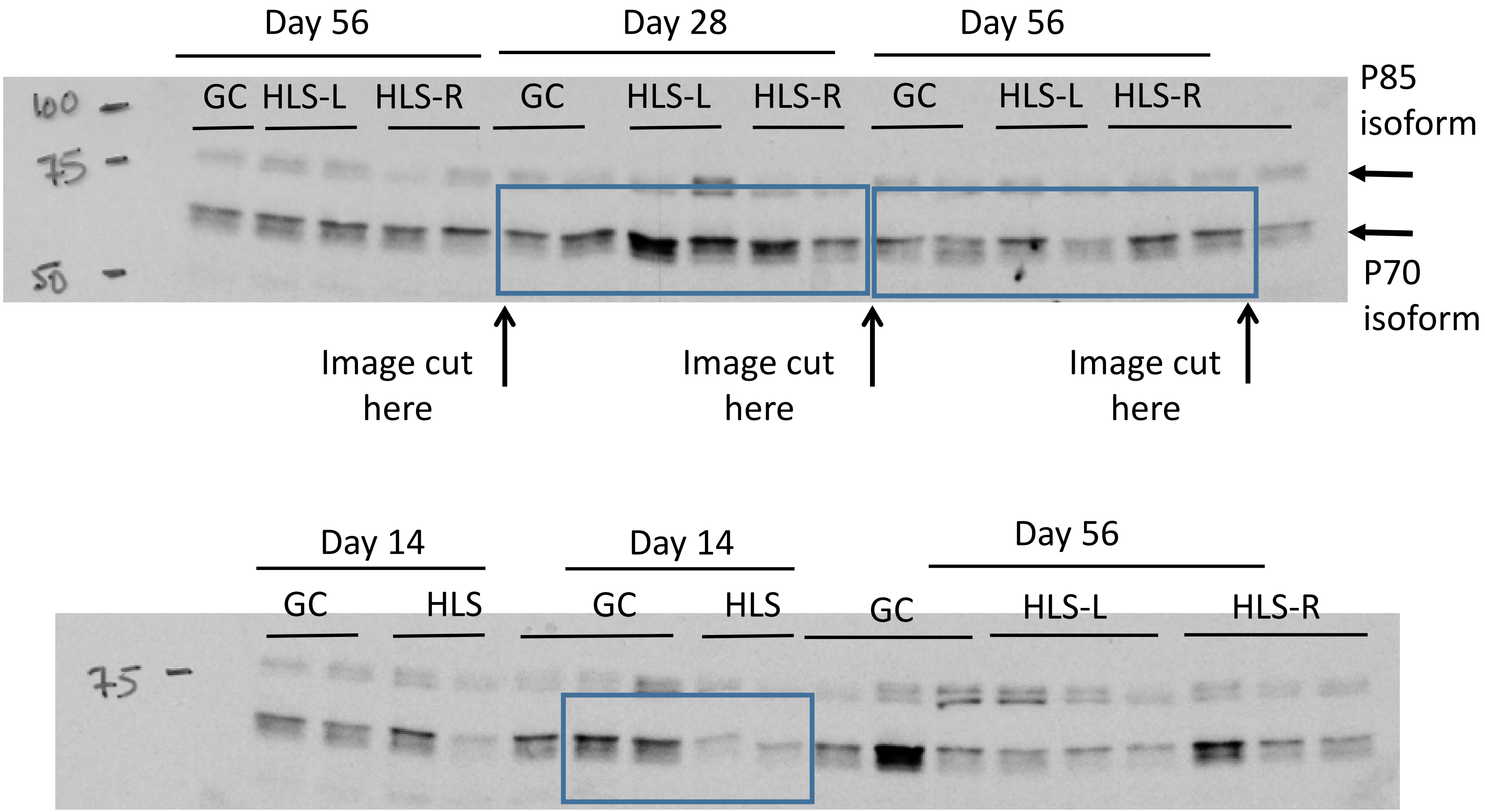

Supplementary Figure 7. Original capture of Figure 6A: S6K1 – Thr389 For presentation purposes, representative blots were obtained from 2 separate gels and blots arranged to match the group order shown in respective bar graphs. All experimental groups for a specific day were obtain from the same gel. Group order was randomized and not always run in the same order to compensate for differences in any differences in transfer across the gel and to blind the technician to group identification. Blots were selected to best represent the overall average for a given experimental group and for each specific day.

Supplementary Table 1.

Antibody verification:

All antibodies used in the current study are listed in the Antibody Registry at: https://antibodyregistry.org/. In addition, the following information is provided regarding validation of respective antibodies used for Western blot analysis:

Ser65-phosphorylated 4E-BP1 (#9451; Cell Signaling Technology (CST), Boston, MA); this antibody detects endogenous levels of 4E-BP1 when phosphorylated at serine 65[1].

Total 4E-BP1, (gift of Scot Kimball, Penn State College Medicine); no band on Western blot for the various phosphorylated and nonphosphorylated forms of the protein in liver from 4E-BP1 knockout mice[2].

Thr389- phosphorylated S6K1 (#9205; CST); siRNA reduced insulin-induced expression of Thr389 phosphorylation of S6K1[3].

Total S6K1 (#SC-230; Santa Cruz Biotechnologies, Dallas, TX); no band on Western blot of cells treated with siRNA targeting S6K1[4].

Total S6 (#2217; CST) and Ser240/244-phosphorylated S6 (#2215; CST); overexpression or knockdown of protein using shRNA lentivirus increases or decreases, respectively, the relative abundance of total and phosphorylated S6 protein in cells [5].

Puromycin (#EQ001; Kerafast); treatment of cells with IGF-I and/or leucine increases total puryomycin incorporation into cellular proteins that is comparable to 35S-methionine incorporation[6].

References for antibody validation:

1. Wang, X., Li, W., Parra, J.L., Beugnet, A. & Proud, C.G. The C terminus of initiation factor 4E-binding protein 1 contains multiple regulatory features that influence its function and phosphorylation. *Mol Cell Biol* **23**, 1546-57 (2003).
2. Dennis, M.D., Kimball, S.R. & Jefferson, L.S. Mechanistic target of rapamycin complex 1 (mTORC1)-mediated phosphorylation is governed by competition between substrates for interaction with raptor. *J Biol Chem* **288**, 10-9 (2013).
3. Julien, L.A., Carriere, A., Moreau, J. & Roux, P.P. mTORC1-activated S6K1 phosphorylates Rictor on threonine 1135 and regulates mTORC2 signaling. *Mol Cell Biol* **30**, 908-21 (2010).
4. Rosner, M. & Hengstschlager, M. Nucleocytoplasmic localization of p70 S6K1, but not of its isoforms p85 and p31, is regulated by TSC2/mTOR. *Oncogene* **30**, 4509-22 (2011).
5. Chen, B. *et al.* Hyperphosphorylation of ribosomal protein S6 predicts unfavorable clinical survival in non-small cell lung cancer. *J Exp Clin Cancer Res* **34**, 126 (2015).
6. Kelleher, A.R., Kimball, S.R., Dennis, M.D., Schilder, R.J. & Jefferson, L.S. The mTORC1 signaling repressors REDD1/2 are rapidly induced and activation of p70S6K1 by leucine is defective in skeletal muscle of an immobilized rat hindlimb. *Am J Physiol Endocrinol Metab* **304**, E229-36 (2013).
