# Supplementary material for: Mechanical loading recovers bone but not muscle lost during unloading
Source: NPJ Microgravity. 2020 Dec 3;6:36. doi: 10.1038/s41526-020-00126-4 (PMC7712877; doi:10.1038/s41526-020-00126-4)
Supplement: Supplementary file 2 — Reporting Summary [file 41526_2020_126_MOESM2_ESM.pdf]

nature research

Corresponding author(s): Henry J. Donahue

Last updated by author(s): Oct 10, 2020

Reporting Summary

Nature Research wishes to improve the reproducibility of the work that we publish. This form provides structure for consistency and transparency in reporting. For further information on Nature Research policies, see our Editorial Policies and the Editorial Policy Checklist.

Statistics

For all statistical analyses, confirm that the following items are present in the figure legend, table legend, main text, or Methods section.

n/a	Confirmed	
☐	☒	The exact sample size (*n*) for each experimental group/condition, given as a discrete number and unit of measurement
☐	☒	A statement on whether measurements were taken from distinct samples or whether the same sample was measured repeatedly
☐	☒	The statistical test(s) used AND whether they are one- or two-sided<br>*Only common tests should be described solely by name; describe more complex techniques in the Methods section.*
☒	☐	A description of all covariates tested
☒	☐	A description of any assumptions or corrections, such as tests of normality and adjustment for multiple comparisons
☒	☐	A full description of the statistical parameters including central tendency (e.g. means) or other basic estimates (e.g. regression coefficient) AND variation (e.g. standard deviation) or associated estimates of uncertainty (e.g. confidence intervals)
☒	☐	For null hypothesis testing, the test statistic (e.g. *F*, *t*, *r*) with confidence intervals, effect sizes, degrees of freedom and *P* value noted<br>*Give P values as exact values whenever suitable.*
☒	☐	For Bayesian analysis, information on the choice of priors and Markov chain Monte Carlo settings
☒	☐	For hierarchical and complex designs, identification of the appropriate level for tests and full reporting of outcomes
☒	☐	Estimates of effect sizes (e.g. Cohen's *d*, Pearson's *r*), indicating how they were calculated

Our web collection on statistics for biologists contains articles on many of the points above.

Software and code

Policy information about availability of computer code

Data collection	nN software was used.
Data analysis	Data were analyzed with GraphPad Prism (La Jolla, CA)

For manuscripts utilizing custom algorithms or software that are central to the research but not yet described in published literature, software must be made available to editors and reviewers. We strongly encourage code deposition in a community repository (e.g. GitHub). See the Nature Research guidelines for submitting code & software for further information.

Data

Policy information about availability of data

All manuscripts must include a data availability statement. This statement should provide the following information, where applicable:

- Accession codes, unique identifiers, or web links for publicly available datasets
- A list of figures that have associated raw data
- A description of any restrictions on data availability

All data generated or analyzed during this study are included in this published article or if not are available from the corresponding author on reasonable request.

Field-specific reporting

Please select the one below that is the best fit for your research. If you are not sure, read the appropriate sections before making your selection.

☒ Life sciences ☐ Behavioural & social sciences ☐ Ecological, evolutionary & environmental sciences

For a reference copy of the document with all sections, see nature.com/documents/nr-reporting-summary-flat.pdf

Life sciences study design

All studies must disclose on these points even when the disclosure is negative.

Sample size	Sample size based on previous studies from our group.
Data exclusions	No data excluded
Replication	All attempts at replication were succesful
Randomization	Mice were randomly chosen and placed in experimental groups.
Blinding	The investigators were blinded when randomly placing mice in groups

Reporting for specific materials, systems and methods

We require information from authors about some types of materials, experimental systems and methods used in many studies. Here, indicate whether each material, system or method listed is relevant to your study. If you are not sure if a list item applies to your research, read the appropriate section before selecting a response.

Materials & experimental systems

n/a	Involved in the study	
☐	☒	Antibodies
☒	☐	Eukaryotic cell lines
☐	☐	Palaeontology and archaeology
☐	☒	Animals and other organisms
☒	☐	Human research participants
☒	☐	Clinical data
☒	☐	Dual use research of concern

Methods

n/a	Involved in the study	
☒	☐	ChIP-seq
☒	☐	Flow cytometry
☒	☐	MRI-based neuroimaging

Antibodies

Antibodies used

Western analysis was performed in a blinded manner using antibodies directed against Ser65-phosphorylated 4E-BP1 (#9451; Cell Signaling Technology (CST), Boston, MA), total 4E-BP1, (gift of Scot Kimball, Penn State College Medicine), Thr389- phosphorylated S6K1 (#9205; CST), and total S6K1 (#SC-230; Santa Cruz Biotechnologies, Dallas, TX), Ser240/244-phosphorylated S6 (#2215; CST), and total S6 (#2217; CST).

Validation

All antibodies used in the current study are listed in the Antibody Registry at: https://antibodyregistry.org/. In addition, the following information is provided regarding validation of respective antibodies used for Western blot analysis:
Ser65-phosphorylated 4E-BP1 (#9451; Cell Signaling Technology (CST), Boston, MA); this antibody detects endogenous levels of 4E-BP1 when phosphorylated at serine 651.
Total 4E-BP1, (gift of Scot Kimball, Penn State College Medicine); no band on Western blot for the various phosphorylated and nonphosphorylated forms of the protein in liver from 4E-BP1 knockout mice2.
Thr389- phosphorylated S6K1 (#9205; CST); siRNA reduced insulin-induced expression of Thr389 phosphorylation of S6K13.
Total S6K1 (#SC-230; Santa Cruz Biotechnologies, Dallas, TX); no band on Western blot of cells treated with siRNA targeting S6K14.
Total S6 (#2217; CST) and Ser240/244-phosphorylated S6 (#2215; CST); overexpression or knockdown of protein using shRNA lentivirus increases or decreases, respectively, the relative abundance of total and phosphorylated S6 protein in cells 5.
Puromycin (#EQ001; Kerafast); treatment of cells with IGF-I and/or leucine increases total puryomycin incorporation into cellular proteins that is comparable to 35S-methionine incorporation6.
References for antibody validation:

1. Wang, X., Li, W., Parra, J.L., Beugnet, A. & Proud, C.G. The C terminus of initiation factor 4E-binding protein 1 contains multiple regulatory features that influence its function and phosphorylation. Mol Cell Biol 23, 1546-57 (2003).
2. Dennis, M.D., Kimball, S.R. & Jefferson, L.S. Mechanistic target of rapamycin complex 1 (mTORC1)-mediated phosphorylation is governed by competition between substrates for interaction with raptor. J Biol Chem 288, 10-9 (2013).
3. Julien, L.A., Carriere, A., Moreau, J. & Roux, P.P. mTORC1-activated S6K1 phosphorylates Rictor on threonine 1135 and regulates mTORC2 signaling. Mol Cell Biol 30, 908-21 (2010).
4. Rosner, M. & Hengstschlager, M. Nucleocytoplasmic localization of p70 S6K1, but not of its isoforms p85 and p31, is regulated by

TSC2/mTOR. Oncogene 30, 4509-22 (2011).
5. Chen, B. et al. Hyperphosphorylation of ribosomal protein S6 predicts unfavorable clinical survival in non-small cell lung cancer. J Exp Clin Cancer Res 34, 126 (2015).
6. Kelleher, A.R., Kimball, S.R., Dennis, M.D., Schilder, R.J. & Jefferson, L.S. The mTORC1 signaling repressors REDD1/2 are rapidly induced and activation of p70S6K1 by leucine is defective in skeletal muscle of an immobilized rat hindlimb. Am J Physiol Endocrinol Metab 304, E229-36 (2013).

Palaeontology and Archaeology

Specimen provenance

Provide provenance information for specimens and describe permits that were obtained for the work (including the name of the issuing authority, the date of issue, and any identifying information).

Specimen deposition

Indicate where the specimens have been deposited to permit free access by other researchers.

Dating methods

If new dates are provided, describe how they were obtained (e.g. collection, storage, sample pretreatment and measurement), where they were obtained (i.e. lab name), the calibration program and the protocol for quality assurance OR state that no new dates are provided.

☐ Tick this box to confirm that the raw and calibrated dates are available in the paper or in Supplementary Information.

Ethics oversight

Identify the organization(s) that approved or provided guidance on the study protocol, OR state that no ethical approval or guidance was required and explain why not.

Note that full information on the approval of the study protocol must also be provided in the manuscript.

Animals and other organisms

Policy information about studies involving animals; ARRIVE guidelines recommended for reporting animal research

Laboratory animals

Male C57BL/6J mice (Jackson Laboratories, Bar Harbor, ME, USA) were used for all experiments. All mice used were approximately 105 days (15 weeks) old ± 3 days and thus skeletally mature on day 0 (start of experiment).

Wild animals

Provide details on animals observed in or captured in the field; report species, sex and age where possible. Describe how animals were caught and transported and what happened to captive animals after the study (if killed, explain why and describe method; if released, say where and when) OR state that the study did not involve wild animals.

Field-collected samples

For laboratory work with field-collected samples, describe all relevant parameters such as housing, maintenance, temperature, photoperiod and end-of-experiment protocol OR state that the study did not involve samples collected from the field.

Ethics oversight

All procedures followed NIH guidelines for experimental animals, and were approved by the Penn State College of Medicine Institutional Animal Care and Use Committee (Protocol # 46451).

Note that full information on the approval of the study protocol must also be provided in the manuscript.
